# Unusual case of non-sexual Mpox transmission in a heterosexual woman

**DOI:** 10.1007/s15010-025-02550-8

**Published:** 2025-05-16

**Authors:** Marc Biller, Julia Lais, Stefan Esser, Stefanie Sammet

**Affiliations:** Department of Dermatology and Venereology, HIV Outpatient Clinic, University Hospital Essen, University Duisburg-Essen, Essen, Germany

**Keywords:** MPOX, Heterosexual woman, Non-sexual transmission

## Abstract

Monkeypox (MPOX) is becoming an important differential diagnosis in non-endemic regions, particularly in patients with atypical dermatologic lesions and systemic symptoms. Although it is more commonly associated with males, it is important to consider MPOX in females, as its presentation may be misattributed to more common infections. This article describes a 28-year-old heterosexual female with cutaneous lesions and systemic symptoms that were initially thought to be a localized infection. Further evaluation confirmed MPOX with a bacterial superinfection. The patient had no sexual but close contact with a confirmed case of MPOX. Clinicians must remain vigilant to recognize the broad spectrum of MPOX presentations and ensure timely diagnosis, isolation, and preventive measures to limit transmission.

The increasing prevalence of monkeypox (MPOX) outside endemic regions, particularly in Europe and North America, has led to an increase in cases, which are often transmitted through sexual contact among men who have sex with men (MSM) [1]. However, diagnosis is difficult as MPOX can occur in a variety of dermatological forms. This image describes an atypical case of MPOX in a heterosexual woman, highlighting an unusual presentation and route of transmission.

A 28-year-old heterosexual woman presented with a 4 cm inflamed nodule on her chin, accompanied by fever, swollen lymph nodes, body aches, and general malaise. The lesion was initially misdiagnosed as a localized skin infection (Fig. 1). The patient reported close physical contact, including “cuddling,” with a confirmed MPOX case at a birthday party in Berlin, although no sexual contact occurred. Despite surgical drainage and antibiotic treatment, the lesion did not resolve, leading the patient to seek care in our outpatient clinic (Fig. 2). A second, smallpox-like lesion on her right forearm raised suspicion of MPOX (Fig. 3). Swab samples confirmed MPOX infection (clade II) via a clade-specific qPCR assay (LightMix^®^ Modular Monkeypoxvirus Kit, TIB Molbiol), with a Ct value of 14.55. A concurrent bacterial superinfection (impetigo contagiosa) was also diagnosed, and clade differentiation was based on the method described by Li et al. [2].

**Fig. 1:**
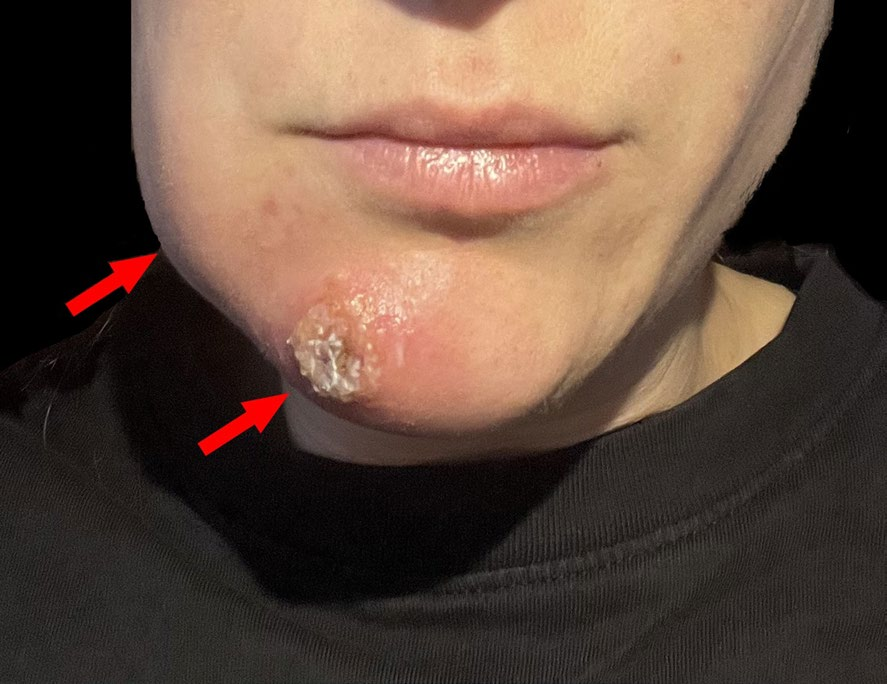
Red arrows highlight swollen lymph nodes and primary skin lesion. This image was captured prior to the patient’s first hospital visit

**Fig. 2:**
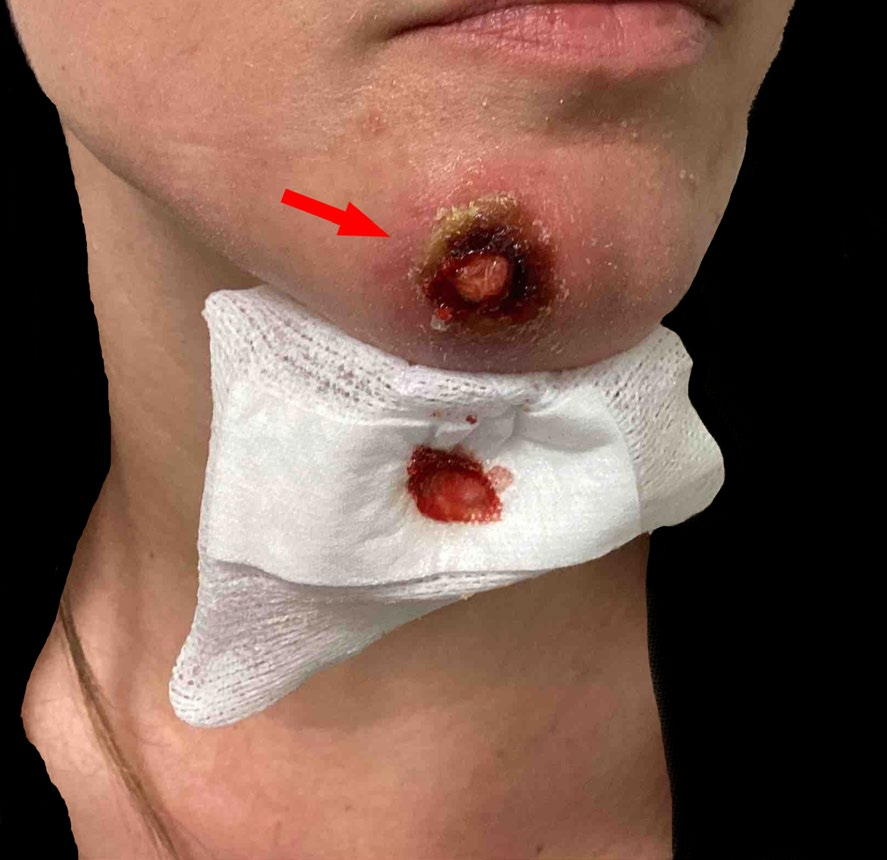
The patient presented to our outpatient clinic six days later in this condition. The red arrow points to the wound following abscess drainage

**Fig. 3:**
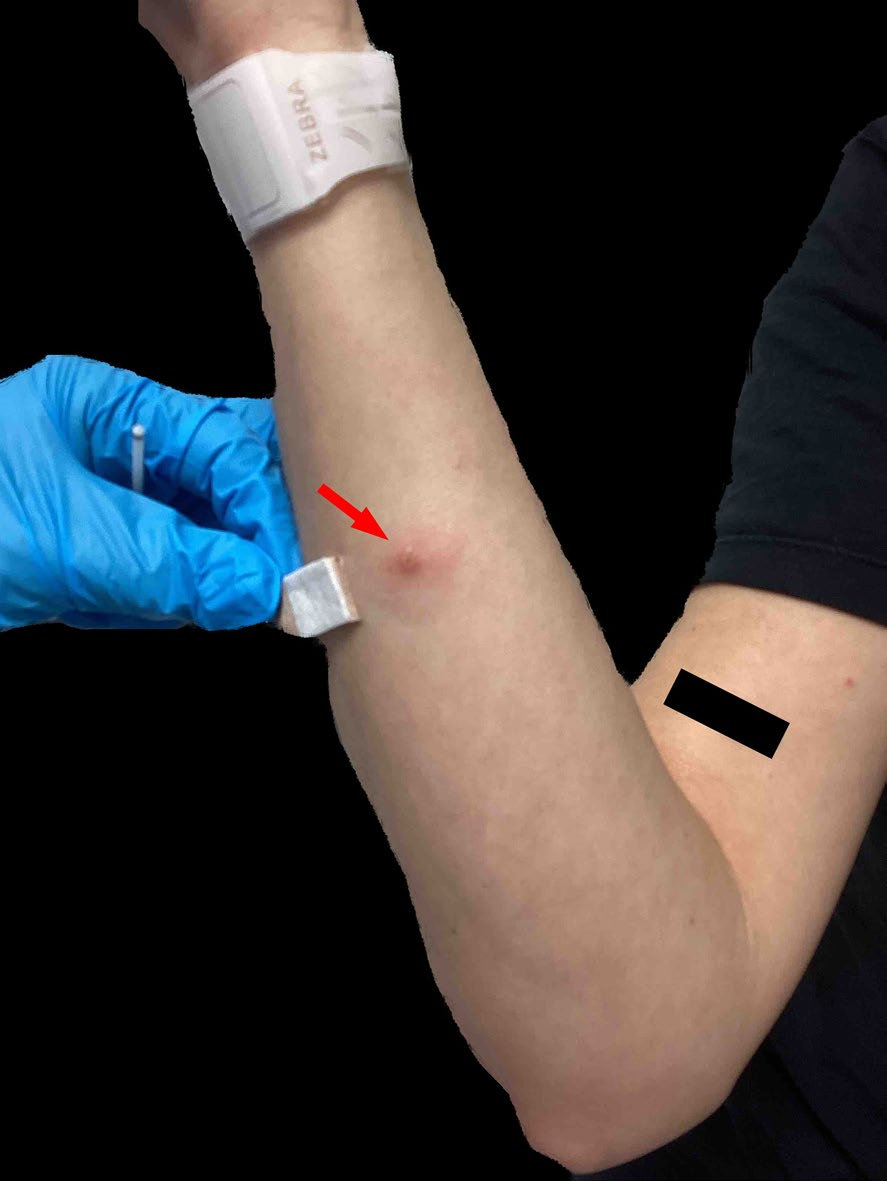
The red arrow indicates smallpox-like lesions observed during the physical examination upon the patient’s presentation at our outpatient clinic

MPOX is often underrecognized in women in non-endemic regions, partly due to the focus on men who have sex with men (MSM) while the 2022 outbreak, which shaped clinical awareness [3, 4]. Only 3.8% of U.S. cases during that outbreak were reported among cisgender women, underscoring a gender disparity in recognition [5]. This case emphasizes the need for clinicians to consider atypical MPOX presentations, especially in patients with systemic symptoms and non-genital lesions. It also highlights the importance of a detailed patient history, diagnostic testing, early isolation, and vaccination strategies to prevent further transmission (Fig. 4).

**Fig. 4:**
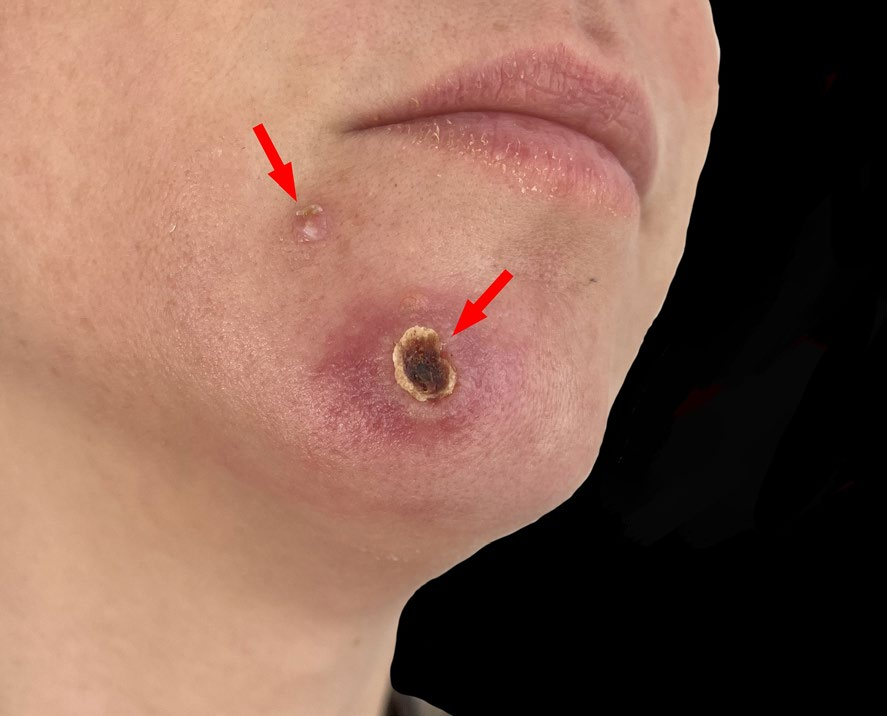
Significant improvement in both the skin lesions and overall symptoms after 10 days. The red arrows highlight the healing of the smallpox-like lesions and the primary skin lesion

## Data Availability

No datasets were generated or analysed during the current study.
